# Attachment security in companion dogs: adaptation of Ainsworth’s strange situation and classification procedures to dogs and their human caregivers

**DOI:** 10.1080/14616734.2018.1517812

**Published:** 2018-09-24

**Authors:** J. Solomon, A. Beetz, I. Schöberl, N. Gee, K. Kotrschal

**Affiliations:** a Department of Public Health and Primary Care, University of Cambridge, Cambridge, UK; b Institute of Parenting, Adelphi University, Garden City, NY, USA; c Department of Behavioral Biology, Konrad Lorenz Research Station, University of Vienna, Vienna, Austria; d Department of Special Education, Institut für Sonderpädagogische Entwicklungsförderung und Rehabilitation, University of Rostock, Rostock, Germany; e College of Professional and Continuing Education, WALTHAM Centre for Pet Nutrition, Leicestershire, UK

**Keywords:** Attachment, sensitivity, temperament, strange situation, dog

## Abstract

This exploratory study describes the development of a classification system for dogs’ attachment security to caregivers that adheres closely to Ainsworth’s seminal methodology. Fifty-nine adult dogs and caregivers participated in a mildly threatening laboratory encounter with a stranger (TS) and the Strange Situation (SSP). Dog and attachment experts adapted Ainsworth’s classification system  for the behavioral repertoire of the dog. Four potentially comparable patterns of attachment were identified. The proportions of secure and insecure classifications (61% and 39%) were similar to those found in human toddlers. Caregivers’ sensitivity to their dogs during the TS procedure significantly differentiated dogs with secure vs. insecure classifications Lower scores on the Active/excited personality scale on the Monash Canine Personality Questionnaire-Revised (MCPQ-R) also were related to secure classification. This system now makes it possible to compare directly the effects of human and dog attachment patterns on the health and emotional well-being of humans and dogs.

The dog–human relationship has become an important focus of investigation across diverse fields, including behavioral evolution and comparative cognition, veterinary science, and clinical and educational interventions with children and adults. From the human perspective, researchers have found improved outcomes for autism-spectrum disorders, medical diagnoses, and child and adult behavioral and emotional health (Nimer & Lundahl, 2015; Payne, Bennett, & McGreevy, 2015).The dog–human bond also has benefits for the dog, including stress reduction mediated by cortisol and oxytocin regulation (Kotrschal, 2016, 2018) and improved learning and resource acquisition in the company of a trusted owner (Horn, Range, & Huber, 2013; Huber, 2016).

A number of investigators have drawn parallels between infant–parent attachment and the bond between dogs and their human caregivers (eg Archer, 1997; Nagasawa, Mogi, & Kikusui, 2009) based explicitly on Bowlby’s ethological theory of attachment (Bowlby, 1969/1982). A variety of self-report measures assessing owners’ experience of protection and comfort with their dog have been developed, as well as assessments of dog owners’ mental representation of their attachment to the dog (Beetz et al., 2014; Dwyer, Bennett, & Coleman, 2006; Kurdek, 2009; Schöberl, Beetz, Solomon, Gee, & Kotrschal, 2016; Zilca-Mano, Mikulincer, & Shaver, 2011) but only Beetz and colleagues have attempted to validate this based on observations of the relationship.

Focusing on the dog’s side of the relationship, investigators have adapted the Strange Situation Procedure (SSP) (Ainsworth, Blehar, Waters, & Wall, 1978) to observe dogs’ secure base behavior with their caregivers. Topál, Miklósi, Csányi, and Dóka (1998) were the first to explore the suitability of the SSP for studying dog–human attachments. Using behavioral coding and frequency counts, they found that dogs preferentially directed attachment behaviors, such as approach and contact seeking, to their caregivers following separations and played and explored more in the presence of the caregiver than a stranger. Prato-Previde, Custance, Spezio, and Sabatini (2003) established an even closer correspondence between dogs and infants in the SSP by coding an expanded range of attachment behaviors and looking more closely at indicators of the secure base effect, such as depressed play during separation. Recently, Rehn and Keeling (2016) acknowledged a growing consensus among animal researchers that dogs form attachment bonds to their owners, based largely on studies of dogs’ secure base behavior in the SSP. They highlighted the identification of attachment patterns, akin to those captured in human studies, as “the next step in anthrozoology research.”

The current exploratory study was designed to contribute to this line of research by adapting Ainsworth’s classification system for infant behavior in the SSP to identify and validate qualitative differences in patterns of dog attachment to their human caregivers. Our first aim is to provide a basis for a standardized measure to describe the dog–caregiver bond, facilitating research into whether patterns of dog–human attachment reflect the history of interaction with the caregiver and have effects on well-being similar to those found for human infants with secure attachments. In infants and young children, secure attachments predict social competence, stress reduction, effective executive functioning and stress regulation, capacity for cognitive complexity, and sensitive parenting many years later (Sroufe, Egeland, Carlson, & Collins, 2005; Weinfield, Sroufe, Egeland, & Carlson, 2008). The reverse is true for the insecure groups, with the disorganized pattern being most clearly associated with behavioral and mental health outcomes in the clinical range (Rutter, Kreppner, & Sonuga-Barke, 2009). Only the disorganized pattern has shown any systematic relation to temperamental or genetic differences among babies (Fearon et al., 2006; Spangler, 2011). Our second aim is to broaden the base of comparative attachment studies both for insights it may contribute to our understanding of the evolution of attachments, including human attachment. The dog is particularly well-suited to this purpose because it is one of the few species outside the primate order that is capable of complex, sustained mutuality in interaction with humans (Kotrschal, 2016, 2018).

## Theoretical and methodological problems

Attachment and human development specialists may question this expansion of the attachment research paradigm, given the evolutionary gulf between humans and dogs and the substantial interspecies differences in behavioral repertoire and cognitive and emotional function. It is interesting to note, however, that there are historical links between attachment theory and observations of attachment behavior in dogs. It is well known that attachment theory originated in Bowlby’s interest in the effect of separating children from their mothers and that he was strongly influenced by ethological studies of the effects of separation from the mother of primate infants. Bowlby was also well aware of research of the time on attachment behavior in dogs, as illustrated by the following quotation:

“Lorenz has recorded that ‘the most sensitive and the most trusting dogs refuse to recognize their masters when they come to fetch them after a period of absence’” (329); “At parting she became desperate; and in the weeks following this second separation she became an unruly delinquent dog. She roamed restlessly about the district, was no longer house-trained, and refused to obey anybody. She also became increasingly ferocious” (Bowlby, 1961, pp. 329).^1^

Presuming that dog–human attachment can be accommodated by ethological attachment theory, what characteristics in the dog permit the development of a cross-species bond? As reviewed recently by Buttner (2016) and Kotrschal (2016, 2018), a growing body of empirical research demonstrates the extraordinary social competence of dogs in interaction with humans which makes relationship possible, including the use of human communication signals to find hidden objects, social learning and imitation, perspective taking, and some forms of empathy. These skills are understood to reflect both the dog’s evolutionary origin in the complex social systems of the wolf (Range & Viryani, 2015) and 35,000 years of adaptation by domestication (Frantz et al., 2016; Thalmann et al., 2013; Wang et al., 2013). Further, the human-adapted social competence of dogs is seen as supported by neurological pathways and neurohormones, such as oxytocin, common to mammals, including humans and other primates (Goodson, 2005; O´Connell and Hoffman 2012; Nagasawa et al., 2015). In sum, these features appear to permit the expansion of sensitive socialization periods in the dog, facilitate acceptance of human guidance and dominance, readiness to read human social cues, and ability to produce enhanced social cues, such as prolonged eye-to-eye gaze and tail wagging, all of which facilitate the level of reciprocity required to establish and maintain an attachment bond (Buttner, 2016).

Dog–human attachment is not necessarily entirely identical to infant–parent attachment, however. In the wild, dog and wolf pups within the first few weeks of life, retreat to the maternal den when threatened; maintaining persistent proximity to the caregiver, the common pattern in primates, may be unnecessary or even counter-productive. Further, the pups appear to transition quickly after weaning (at approximately 7 weeks) from parental dependence to complex affiliative bonds and dominance interactions with other pack members (Spotte, 2012). Separation distress when separated from mother and littermates begins at about 3 weeks of age but drops off considerably by 16 weeks (Scott, 1963). In addition, under some circumstances, dogs also have been reported to rapidly transfer their attachment behavior to a new human partner (Gàcsi, Topàl, Miklòsi, Dòka, & Csànyi, 2001; Valsecchi, Previde, Accorsi, & Fallani, 2010). Thus, in contradiction to Lorenz’s observations, there is room to challenge the notion that dogs establish long-term, stable attachments to particular individuals, which is an essential component of the human attachment system (Ainsworth, 1989, 1991). There likely are other important ontological differences, variations among breeds of dogs, as well as differences at the level of biological mechanisms in the manifestation of dog attachments that have yet to be determined. Finally, since humans both perceive their pet dogs as attachment figures and conceive of themselves as caregivers (Beetz et al., 2014; Dwyer et al., 2006; Kurdek, 2009; Schöberl et al., 2012) we may find, ultimately, that dog–human attachments, especially those between adult dogs and their caregivers, share features of human friendships and adult pair bonds, in which attachment is but one component in a multi-faceted relationship (Ainsworth, 1989).

We also may ask whether it is appropriate to use the SSP to assess important aspects of the dog–human bond. We propose that, for heuristic purposes we can be guided by Bowlby’s ethological attachment theory, which dictates the type of behavior (if not the specific behaviors) and the circumstances in which we should expect to see them (some of which may be common among species, others not). Thus, the question of appropriateness ought to be based on whether the *organization* of behavior of the majority of dogs with their caregivers in the SSP conforms to theoretical predictions. The hallmark of attachment is the display of behaviors (eg distress vocalizations, greetings, following and approach) that bring the one attached to a specific individual – the attachment figure – or bring that figure to himself (Ainsworth, 1991; Bowlby, 1969/1982). Theory predicts that attachment behavior will be activated (evoked) by threatening circumstances, many of which are common among vertebrates, such as novelty, looming objects, loud sounds (Hinde, 1966), as well as separation from the attachment figure. For human infants, the attachment system is terminated (assuaged) by proximity and contact with the attachment figure. This terminating condition is likely to pertain for all mammals, but for nesting or den-living species, stimuli, such as familiar locations, smells, and sounds may be as or more potent than they would be for human infants (Hofer, 1994). Lastly, under conditions of novelty, such as an unfamiliar place and unfamiliar persons, the presence of the attachment figure should support exploration and play – the secure base phenomenon. When the infant is alarmed, play and exploration are expected to be supplanted by attachment behaviors and to reemerge once safety is signaled by the return of the attachment figure.

The SSP protocol was designed by Ainsworth to demonstrate the theoretically expected shifts in behavior described above. It exposes the infant to a graduated series of stressors, ie a novel environment with mother present, a strange adult, first with and later without mother present, and two separations and reunions from mother. Researchers interested in dog–human attachment in the main have concluded that the organization of dog behavior translates readily to the structure of the SSP and infant–parent behavior. Although knowledge of dog communication signals is necessary for accurate judgments, it is not difficult, after all, to equate the enthusiastic tail wagging of the dog on reunion with the smiles and greeting vocalizations of the human infant nor whining, barking, and scratching at the door with search and calling. And, although few dogs insist on being held in arms after reunion, as infants do, dogs do actively and persistently insist on physical contact, for example by placing their head in the lap of the caregiver.

Ainsworth emphasized that the presence of attachment-relevant behaviors in the SSP do not in themselves provide a demonstration of the existence of the bond. This bond is simply assumed in studies of home-reared human infants, an initially controversial assumption now supported by considerable research, including observations of non-attached children in the SSP (Zeanah, Smyke, Koga, & Carlson, 2005). Investigators of dog–human attachment, who must be especially cautious about unwarranted inferences regarding the inner states of their subjects, and who have been interested mainly in demonstrating the existence of the bond, have taken pains to manipulate the parameters of the protocol to address ambiguities. These efforts include modifications of the order, timing, and format of the episodes of the SSP, such as adding separation and reunion episodes, counterbalancing the order of episodes, and restricting the behavior of the caregiver (eg Palmer & Custance, 2008; Rehn, McGowan, & Keeling, 2013; Taggert, 2010). The purpose of such changes has been to clarify dogs’ preference for interaction and physical contact with the caregiver over the stranger and explain shifts in exploration related to separations and reunion. The results of these manipulations have largely supported the underlying premises of the original SSP format when dogs rather than babies are subjects. Analogously, when preschool age children are seen in the SSP, changes in the length or number of separations do not appear to interfere significantly with the ability of well-trained observers to make reliable judgments of classification group (Cassidy, Marvin, 1992; Main & Cassidy, 1988).

## Identification and validation of attachment patterns

By 12 months of age, the quality, or security of the infant–parent bond can be demonstrated in the SSP. Ainsworth found that 1-year-olds who have experienced sensitive and responsive care from the mother show a pattern of behavior in the SSP that is closest to Bowlby’s theoretical expectations. These infants explore in the mother’s presence, seek her out and maintain contact with her following separation, and, once reassured, resume exploration; this pattern is termed “secure.” Ainsworth also described two variant patterns: (1) the *avoidant* pattern, in which the infant persistently averts his gaze and maintains physical distance from mother on reunion, is linked to rejecting and intrusive maternal behavior; and (2) the *resistant* pattern, characterized by the infant’s persistent angry resistance and distress, is associated with unreliable maternal care (Ainsworth et al., 1978). The avoidant and resistant patterns are viewed as insecure in that they demonstrate the infant’s adaptation to negative expectations about the responsiveness of the caregiver. Main and Solomon (1986, 1990) later identified a third insecure pattern of attachment termed disorganized-disoriented, characterized by disruptions in the organization of the standard Ainsworth strategies, such as simultaneous approach-avoidance, direct indices of fear, freezing, stilling, and disorientation. The disorganized pattern is associated with deleterious caregiving experiences, including frightening, helpless, or dissociated maternal behavior (Lyons-Ruth & Jacobvitz, 2016) or major separations (Solomon & George, 1999). This exploratory study tests the hypothesis that similar variations and disruptions of attachment behavior may be found in dogs, also linked to the quality of interaction between dogs and their caregivers.

As yet, no attachment typology comparable to Ainsworth’s is available for the dog, although efforts in that direction have been undertaken. Typically, researchers have made subjective assessments or used cluster analysis of behavior coding to find patterns that appeared to correspond more or less to Ainsworth’s descriptions of security. Insecurity has been defined mainly by high levels of attachment behavior accompanied by signs of separation anxiety (Fallani, Previde, & Valsecchi, 2006; Rehn & Keeling, 2011; Topál et al., 1998; but see Taggert, 2010). These studies suggest that at least some individual differences in patterns of attachment security can be detected in the dog. A parallel line of research documents similarities between caregiver and dog personality or attachment pattern, based on caregiver self-report. These findings have been interpreted to suggest that dogs adapt to the interactive style of their caregivers, as children do to their primary caregiver, although other interpretations are also possible, such as that dog caregivers select breeds whose personality are compatible with their own (Ragatz, Fremouw, Thomas, & McCoy, 2009; Rehn, Lindholm, Keeling, & Forkman, 2014; Schöberl, Wedl, Bauer, Day, Möstl, & Kotrschal, 2012; Siniscalchi, Stipo, & Quaranta, 2013; Wedl, Schöberl, Bauer, Day, & Kotrschal, 2010)

Ainsworth’s work demonstrated that distinguishing coherent patterns in the SSP depends on observations of the subject’s reaction to increasing levels of stress and the way in which they make use of the attachment figure in their recovery from stress during the reunion episodes. These are evinced by differences in the timing, intensity, and persistence of attachment and exploratory behavior across the entirety of the SSP procedure and the observation of defensive behaviors, such as avoidance and resistance. The methodology in previous studies of dog–human attachment has not been well suited to revealing these patterns, however. Changes to the SSP protocol in some studies (eg Palmer & Custance, 2008; Rehn et al., 2013; Taggert, 2010) necessarily would interfere with detecting many distinguishing features of the Ainsworth patterns. In addition, animal researchers typically measure the duration and frequency of behaviors, narrowly defined to maintain observer reliability; the qualitative aspects of the interaction are thereby lost. For example, many animal investigators typically define proximity-seeking “as approach to the caregiver within a fixed circumference,” whereas Ainsworth’s system highlights features, such as who takes the initiative in making contact, actions taken by the infant to maintain physical contact or failures to take such action, and subtle signs of avoidance, discomfort or anger in contact all of which may have an impact on the timing and intensity of proximity seeking. Crucially, the usefulness of behavior coding to detect stable patterns of behavior depends upon extensive and equivalent opportunity and conditions. Putative patterns or classifications based on behavior coding tend to be unstable, however, when tests are repeated or transferred to other contexts (Waters, 1978). In confirmation of this point, two recent studies demonstrate the differential utility of frequency coding and qualitative methods for describing dog behavior depending upon on the context and the variables of interest (McGarrity, Sinn, Thomas, Marti, & Gosling, 2016; Walker, Dale, D’Eath, & Wemelsfelder, 2016). In contrast, Ainsworth’s methodology includes rating of major dimensions of attachment behavior which take both qualitative and contextual factors into account followed by assignment of each case to a best-fitting classification group defined by the pattern of behavior across all the SSP episodes.

For human infants, the demonstration of a link between attachment patterns and variations in caregiver sensitivity to the infant’s attachment signals is the necessary condition for establishing the validity of measures of security (Solomon & George, 2016). Recent infant studies confirm Ainsworth’s original findings that caregiver responsiveness to infant distress is the variable most predictive of infant attachment security (Leerkes, 2011). In this study, we test the proposition that an independent assessment of caregiver sensitivity to the dog under stress will be associated with patterns of attachment behavior in the dog that are morphologically or functionally similar to those of infants. Based on previous studies, it appears that both secure and anxious-resistant patterns will be identifiable. An avoidant pattern reportedly is non-existent or very rare in monkeys (Hinde, 1966) and clear disorganization of attachment behavior has been reported only in a single study of captive chimpanzees with their handlers (Van Ijzendoorn, Bard, Bakermans-Kranenburg, & Ivan, 2008). Both types of behavior, however, are commonly described in the ethological literature and therefore we expect to observe them in dogs, but with what frequency or intensity has yet to be determined. Finally, in the human case, well-designed twin studies have demonstrated little or no effect of temperament variables on the Ainsworth patterns. (Fearon et al., 2006). This may not be the case for dogs, however, which have been bred extensively for temperamental variations (Parker et al., 2004). In this study, therefore, we also evaluate the role that commonly assessed dog temperament variables might play in the development of attachment pattern in this species, without making specific predictions.

## Methods

### Subjects

The subjects were 59 intact, medium sized (15–35 kg) adult (mean age ± SD: 3.96 ± 1.54 years) companion dogs and their human caregivers. Dyads were recruited as part of a larger study on biopsychological factors affecting the caregiver–dog relationship (*N* = 132) (Schöberl, Wedl, Beetz, & Kotrschal, 2017; Schöberl et al., 2016). Approximately, half of the dogs were male (*n* = 28) and half were female (*n* = 31). The caregivers (mean age ± SD: 46.22 ± 10.22 years) comprised about 50% males (*n* = 29) and females (*n* = 30). All dogs were adopted as puppies (mean age ± SD: 9.80 ± 4.17 weeks). Team composition (ie caregiver–dog gender combinations) was counterbalanced (17 female–female pairs; 14 of each of the remaining 3 groups). Mongrels (*n* = 8) and a variety of purebred dogs participated (not more than three dogs of the same breed per team-gender combination).

#### Recruitment

Participants for the larger study were recruited via newspaper, internet, and networks (eg dog training organizations). Only caregiver–dog dyads that completed participation in the larger study were asked to participate in a third meeting, when the SSP was conducted. Caregivers were informed at the start of the study that the general aim was to better understand human–dog relationships and learned prior to the SSP and that this session examined how dogs behave in the presence of a stranger with and without the caregiver present. No mention was made of our particular interest in attachment; caregivers were debriefed at the end of the session. All participants were informed that saliva samples would be taken, that sessions would be videotaped, and that the sessions would be curtailed if the experimenter observed extensive stress signs in caregiver or dog. This did not occur in any of the SSP sessions.

Participants signed two information and consent forms, one for the first and second meeting and one for the third meeting. Data collection was conducted according to the standards of the Code of Ethics of the World Medical Association (Declaration of Helsinki), the EU Directive 2010/63/EU for animal experiments and Uniform Requirements for manuscripts submitted to Biomedical journals and the German society of Psychology (Ethische Richtlinien der DGPs und des BDP). Ethical review for the first and second meeting was done by the animal-welfare committee of the Faculty of Life Sciences, University of Vienna (approval number: 2014–015). Ethical review for the third meeting was done by the German Society for Psychology (Deutsche Gesellschaft für Psychologie), because it occurred later and it was felt that an ethical review independent of the first two sessions would be advantageous.

### Procedures

The larger study involved two laboratory visits approximately two weeks apart to the University of Vienna (Austria) Department of Behavioral Biology tests rooms. All sessions included caregiver questionnaires, structured observations, and self-administered dog and caregiver saliva sampling. Video recording was continuous during all sessions, using a camcorder (Canon Legria-HF-G10) with a wide-angle conversion lens (Canon WD-H58W) fixed to the wall of the test rooms.

During the second visit, the dog was exposed to two episodes with a “Threatening Stranger (TS).” Counterbalanced order of testing, with and without the caregiver present (referred to later as “threat order”) were randomly assigned. Ratings of caregiver sensitivity (below) were based on the TS episodes in which the caregiver remained in the room with the dog. The SSP occurred during the third laboratory visit four weeks to one year later (depending upon caregiver and laboratory availability) at another location from the first two sessions. An unfamiliar testing room was a requirement of the procedure because novelty activates both attachment behavior and exploration (Bowlby, 1969/1982). Attachment classifications and behavior ratings were derived from videotapes of these sessions.

#### “Threatening Stranger” (TS) procedure

Two TS episodes took place approximately 45 and 65 min after the dyad entered the test room. In each TS episode, the dog was tethered to the wall by the caregiver just prior to the entry of an unfamiliar (female) graduate student, dressed in a black coat with a hat and ski mask (just the eyes were visible). The TS entered the room, knocked on the closed door to get the attention of the dog (and another two times if the dog did not react) and stared at the dog’s eyes or face continuously while moving three steps toward the dog, with a 3 s pause between each step. The placement for each step was marked on the floor. The TS left the room after the first threat episode. After the second threat episode, she moved to a distance, removed the costume, approached the dog sideways, yet not too closely, talking in a friendly way and offering pieces of cheese, then left the room. When the caregiver was present he/she was instructed to behave as in similar situations during daily life. When the caregiver was absent, he or she could observe the situation from outside via a monitor. The administrator also observed the procedure from outside during both conditions. Saliva sampling occurred immediately before each TS episode and 15 min after each TS. Cortisol data are reported elsewhere (Schöberl et al., 2016).

#### The Strange Situation Procedure (SSP)

**Figure 1.**
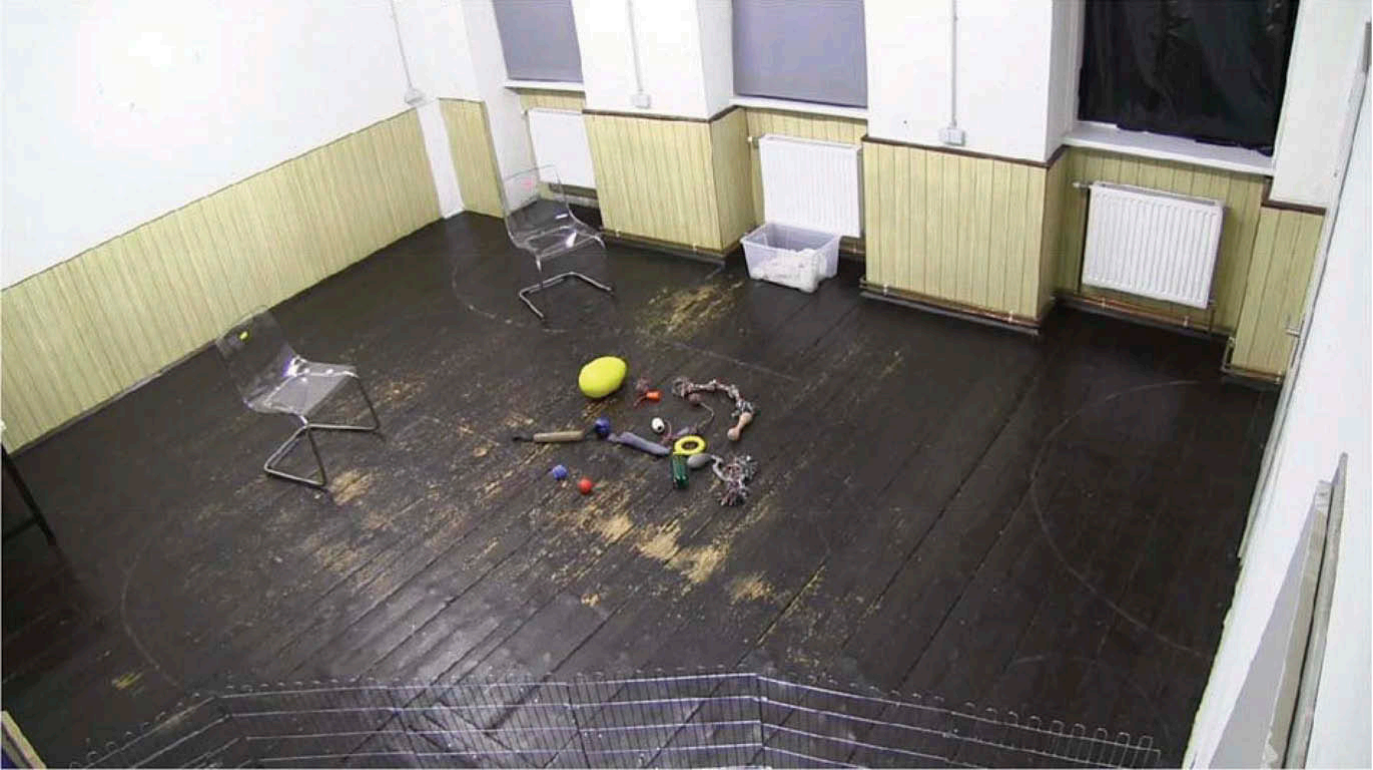
Set-up of the SSP playroom.

This 22-minute procedure took place during the third laboratory visit in a room at another location from the first two sessions. The room set-up is shown in Figure 1. A variety of suitable toys were placed in the center of the room. The procedure was conducted according to the order and timing of the standard eight episodes of Ainsworth’s infant procedure (Ainsworth et al., 1978). Instructions to caregivers, administrators, and the stranger (playmate) were adapted from Solomon (unpublished manuscript). All episodes were three minutes in length (except for Episode 1, the introduction to the room). Caregivers were cued to leave by the stranger, when she was present, or by a mobile phone vibrator, when they were alone with the dog. The caregiver received additional instructions and reminders from the administrator before the session and when he or she was out of the room. The content of the episodes, timing, and key instructions to parents are summarized in Table 1.

**Table 1.** The episodes of the SSP.^a^

Episode number, title and Timing	Description and key caregiver instructions
**Episode 1** *Entry to the playroom* 30 s	Caregiver and dog enter the playroom Administrator shows caregiver where to sit
**Episode 2** *Free play* 3 min	Caregiver and dog only “Act as you might in a similar situation”
**Episode 3** *Introduction of the stranger* 3 min	Stranger enters Silent: 1 min, Stranger chats with caregiver: 1 min Stranger plays: 1 min
**Episode 4** *Separation 1* 3 min or less	Caregiver leaves “Do what you would do usually if you were leaving for a few moments.” Stranger plays with or reassures dog; withdraws at sound of caregiver’s approach
**Episode 5** *Reunion 1* 3 min	Caregiver re-entry Caregiver calls dog’s name and enters, greets, and returns to chair^b^ Stranger leaves quietly.
**Episode 6** *Separation 2* 3 min or less	Caregiver leaves; Dog remains alone “Do what you would usually if you were leaving for a few moments.”
**Episode 7** *Stranger & dog* 3 min, or less	Stranger returns Stranger plays with or reassures dog; withdraws at sound of caregiver’s approach
**Episode 8** *Reunion 2* 3 min	Caregiver re-entry Caregiver calls dog’s name and enters, greets, and returns to chair^b^ Stranger leaves quietly Caregiver is instructed to initiate contact at least once in episode

^a^Adapted from Ainsworth et al. (1978)

^b^Caregivers in this study were asked to remain seated and play with their dogs from there during the reunion. Many caregivers nevertheless chose a more active role.

### Measures

#### Secure base behavior coding

As part of the larger study, caregiver and dog behaviors in the SSP were coded by two individuals using the software The Observer XT (Noldus). Three behaviors were selected a priori for this study to evaluate the overall suitability of procedures to elicit secure base behavior in dogs: (1) duration of independent exploration (dog’s activity is directed toward objects or unmovable aspects of the environment, eg sniffing, visual inspection and oral examination, rubbing mouth/body on object); (2) duration of whimpering (dog utters, soft, intermittent sounds, with closed to slightly opened mouth, maximum break of two seconds); and (3) duration of physical contact with caregiver or stranger (dog nudges caregiver/stranger with its muzzle, dog reaches out with forepaw toward person, dog leans or rubs body or part of body against person including leaping with body contact). These behaviors were similar to those used for the same purpose in a study of captive chimpanzees and their human caregivers in the SSP (van IJzendoorn, Bard, Bakermans-Kranenburg and Ivin (2008). Durations were relative, calculated as “Duration of the event” × 100/Interval (seconds). Inter- and intra-observer reliability was assessed before, during, and after completion of coding. Coder agreement for durations was very high (Kappa = 0.93).

#### Caregiver’s reassuring presence (CRP)

The caregiver’s behavior when in the room with the dog during the TS episode was rated on a 7-point scale. The scale was designed for this study to capture caregiver sensitivity to the dog’s response to the TS. Dog responses ranged from arousal to fear marked by loud barking, frightened withdrawal, or both. The scale was modeled after Ainsworth’s maternal sensitivity scale (Ainsworth et al., 1978) and the “Supportive Presence” scale developed by Matas, Arend, and Sroufe (1978) for use in structured dyadic tasks. Odd points on the scale were anchored with detailed behavioral descriptions. High scores were given for the caregiver’s prompt and consistent responsiveness to the dog, as indicated by visual monitoring and maintaining proximity to the dog, using reassuring voice and physical comforting, as appropriate. Lower scores were given when the caregiver remained at a distance, was inattentive, and/or displayed negative affect, such as embarrassment or irritation, or if they further agitated the dog with their physical interventions. Ratings were completed entirely by one of the authors (IS), a specialist in dog behavior who was blind to the dogs’ attachment classification. She participated with the first author in adapting the maternal sensitivity scales to the developmental sample of 10 dyads (also used to develop the dog attachment classification system). To assess inter-judge rating reliability, the first author provided independent ratings for an additional group of 20 dyads that she had not yet observed in the SSP. Her attachment classification judgments for those dogs were completed four to six months later, by which time she had no specific memory of the caregiver or dog or their behavior during the TS. Agreement on the independent ratings was 89% (weighted Cohen Kappa = 0.78). Only the scores of the blind rater were used in statistical analyses.

#### Monash Canine Personality Questionnaire-Revised (MCPQ-R)

Caregivers described their dog’s temperament and behavior with a 26 item questionnaire developed by Ley, McGreevy, and Bennet, (2009). A recent meta-analysis demonstrated that this measure along with behavior-based measures shows high reliability over time (Fratkin, Sinn, Patali, & Gosling, 2013). The questionnaire was translated to German in cooperation with a bilingual expert. A principal component analysis (PCA) was conducted, resulting in five axes comparable to those developed by Ley et al. (2009), as well as to those recommended by Fratkin et al., 2013): Active/excitable; Obedient/reliable; Insistent/goal directed; Nervous/anxious; Friendly/sociable. Scale reliabilities (Cronbach’s alpha) ranged from .71 to .81.

#### Attachment classification

Classification criteria were adapted from Ainsworth et al. (1978) by the first two authors with consultation by the last author on difficult cases. Every effort was made to establish the comparability of measures, making allowances only for differences in the canine behavioral repertoire. For example, at reunion, secure human infants typically immediately walk or crawl directly to the caregiver, make physical contact on their own initiative by, for example, grabbing the caregiver’s legs; they relax into contact once picked up, and they do not seek release for at least 10 s, usually more. The corresponding criteria for “very active proximity and contact seeking” in the dog included rapid, full approach when the caregiver re-entered the room followed by initiating physical contact with the caregiver, eg touching the caregiver with head or snout, jumping up with paws on the caregiver’s body, or sustained leaning against the caregiver’s body or hands. Proximity and contact typically were maintained or reinstated by following the caregiver to his/her chair, placing the head, snout, or paw above or on the caregiver’s lap or otherwise making physical contact.

Ainsworth’s infant attachment classifications are based on observations of shifts in behavior across all eight episodes of the SSP, with an emphasis on behavior in the reunion episodes. As a first step, the two authors developed preliminary classification guidelines for 10 cases, referred to as the “development sample”: Six of these cases were selected arbitrarily from the beginning, middle, and end of the sample pool and four cases were selected based on administration records that suggested “unusual” behavior. The guidelines were further refined on the next 30 randomly selected cases which were independently classified and then discussed to address areas of disagreement and uncertainty. A final group of 18 cases (31% of the sample) was reserved to provide an estimate of inter-judge reliability based on each judge’s independent (pre-conference) classifications. Classification agreement was 89% (16 of 18 cases; κ = .70).

The final classifications for all dogs in the sample represented consensus judgments with the following exceptions: Working independently, classifiers were unable to reach consensus on group placement for 13 cases (22% of the total sample). Five of these showed an ambivalent pattern of behavior but the judges did not agree on whether the case was insecure or “secure-with-some-ambivalence” (referred to as B4 in the human infant literature). For data analysis, all five were placed in the ambivalent category. The remaining eight cases without consensus were placed in the “Unclassified” category, described below. These cases were dropped in classification related analyses, resulting in 51 cases for those analyses. Criteria for the attachment classifications are summarized below:

##### Secure (Group B)

Dog shows active proximity and contact seeking, ie approaches caregiver promptly at reunion and makes physical contact or signals for contact. Once contact is achieved, the dog does not break contact for at least 10 s. There is little or no gaze aversion or proximity avoidance; there is little or no resistance to contact or interaction. In the pre-separation episodes the dog engages in some independent or social play or exploration. (Unlike human infants, many dogs showed little or no independent exploration or play by the 2nd reunion). Sleeping or lying down *after* proximity or physical contact was sought and achieved at reunion did not disqualify a dog from placement in the secure category unless it was associated with ignoring the overtures or requests of the caregiver. The dog shows some active search, but not necessarily distress, in all separations.

##### Insecure-avoidant (Group A)

Dog shows little tendency to approach, to seek contact, or to follow. Dog turns, looks, or moves away and/or shows lack of response to invitations to approach or interact for the first 30 s of reunion or more. Dog explores the room and objects during pre- and post-separation. There is little active search for caregiver during separations, except when the dog is left alone in the room.

##### Insecure-Ambivalent (Group C)

On reunion, the dog makes strong efforts to maintain physical contact mixed with persistent distress and/or physically intrusive behavior directed toward the caregiver. The dyad is characterized by a degree of conflict regarding physical contact or play activities (eg the dog attempts to maintain contact and is uncooperative with the caregiver’s attempt to encourage play or exploration; or, once proximity is sought by the dog, caregiver actively maintains contact despite the dog’s signals of readiness to explore). In the pre-separation episodes, the dog shows little interest in exploration and/or the playmate and clearly prefers to remain nearby the caregiver. During separations, the dog makes frequent distress vocalizations and shows some active search (though he/she may also remain near the playmate for reassurance).

##### Insecure-disorganized/disoriented (Group D)

Disorganized behavior refers to behavior that is inexplicable or contradictory in the context of interaction with an attachment figure and/or with respect to the organized A, B, or C patterns of attachment. It often manifests as a sudden and marked disruption of ongoing proximity seeking, contact maintaining, avoidance, or contact resistance; if strongly present, disorganized behaviors can make it difficult to perceive any underlying classification. Main and Solomon (1990) differentiated seven broad categories of indices listed here with examples of behavior displayed by dogs in the sample: I. Sequential contradictions, eg strong distress in separation followed by strong avoidance, stilling or freezing immediately upon reunion. II. Simultaneous contradictory behavior, eg approaching the caregiver with head sharply averted; III. Undirected, misdirected, incomplete, and interrupted behavior, eg expressions of fear or distress accompanied by or followed with moves away from (rather than to) the caregiver; attempts to make physical contact are interrupted by rapid withdrawal of a paw; IV. Stereotypies, asymmetrical movements, mistimed movements or anomalous postures, eg unpredictable bouts (bursts) of activity or movement that seem to lack normal preparation time or have a jerky, automaton-like quality, eg persistent, rapid circling of the room without apparent rationale; V. Freezing, stilling, and slowed movements and expressions, often accompanied by a dazed or trance-like expression, eg in a prone posture, staring at an unmoving object for 30 s or more; VI. Indices of apprehension, eg moving behind caregiver without immediate rationale, such as to explore an object; fearful postures or movements; VI. Direct indices of disorganization or disorientation, eg confused sequences of very rapid changes of affect in the first few seconds of reunion, eg rapid movements of approach followed by rapid withdrawal, followed by approach again; apparently “aimless” wandering around the room.

To be classified as disorganized, the dog’s odd or inexplicable behavior should be frequent, extreme, or extensive and more evident in the caregiver’s presence than absence. Particular weight was given to repeated manifestations, the appearance of several different indices of disorganization, and disorganized behavior that accompanied or closely followed the moments of reunion (within 30 s of reunion). Whenever making a disorganized/disoriented classification, classifiers also noted the A, B, or C group that the dog’s attachment behavior most nearly resembled. In infant studies this “best-fitting” alternative is useful for comparisons with data classified only with Ainsworth’s A, B, and C system or to facilitate comparisons with the caregiver’s attachment representation (van IJzendoorn, 1995).

##### Unclassified

The dog’s behavior seems disturbed but it is too ambiguous to classify. For example, it is unclear whether the dog is frequently dissociating in the caregiver’s presence or simply reacting to distant sounds that the coder can’t hear; or, the dog is skittish and circles the room repeatedly, whether or not the caregiver is present, suggesting a neurological or compulsive condition; or, the dog’s greetings and approaches to the caregiver are markedly lethargic, possibly suggesting depression or physical illness.

### Statistical analyses

SPSS 20.0 was used for statistical analyses. To determine whether secure base behavior changed as expected across the SSP episodes, the durations of independent dog exploration, body contact with caregiver or stranger, and whimpering were entered into separate ANOVAS with repeated measures analyses. Data were available for five points during the SSP – Pre-separation (episodes 1, 2, and 3); Separation 1 (stranger only in the room); Reunion 1, Separation 2 (episodes 6 – dog alone, and 7 – stranger reentry, combined); and Reunion 2. Significant main effects were followed up with planned, paired sample *t*-tests. The Holm’s Sequential Bonferroni Procedure was used to adjust alpha for these *t*-tests because of the increased risk of Type I error when there are multiple comparisons. The overall target alpha was two-tailed, *p *= .05, but the assessment of significance for any one comparison is adjusted according to the rank of its *p*-value with respect to the other comparisons.^1^ Cohen’s effect size was also calculated for all univariate statistics, with *d* = 0.2 indicating a small effect, *d* = 0.5 a medium effect and *d* = 0.8 a large effect (Cohen, 1988).

To determine whether dog attachment classifications primarily reflected the caregiver’s sensitivity and/or was influenced by other factors, a Generalized Linear Model (GLM) with binomial distribution was calculated. For the purpose of analysis, avoidant, ambivalent, and disorganized groups were combined into a single, insecure group. Therefore, secure vs. insecure classification was used as the dependent variable. Covariates comprised caregiver reassuring presence (CRP), gender and age (in years) of caregivers and gender and age of dogs, and the five MCPQ-R scales. The Akaike information criterion (AIC) was used to decide the best-fit model. Following Schöberl et al. (2016), any terms with *p* < 0.1 were permitted to remain in the final model, however only terms with *p* < 0.05 were considered as having a significant influence on the dependent variable. Excluded terms were re-entered one by one into the final model to confirm that they did not explain a significant proportion of the variation . In all cases, the alpha level stated (*p* < 0.05) is two-tailed.^2^ All *t* and *F* statistics also were evaluated using Cohn’s *d* (as a measure of effect size).^3^

## Results

### Demonstration of secure base effects

**Figure 2.**
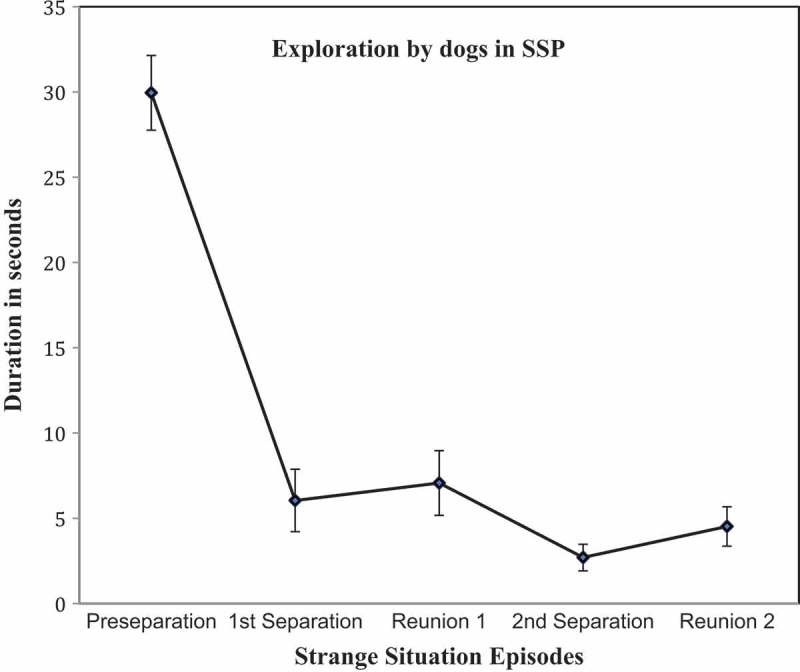
Exploration of the room by dogs in the episodes of the SSP (Mean ± SE).

**Figure 3.**
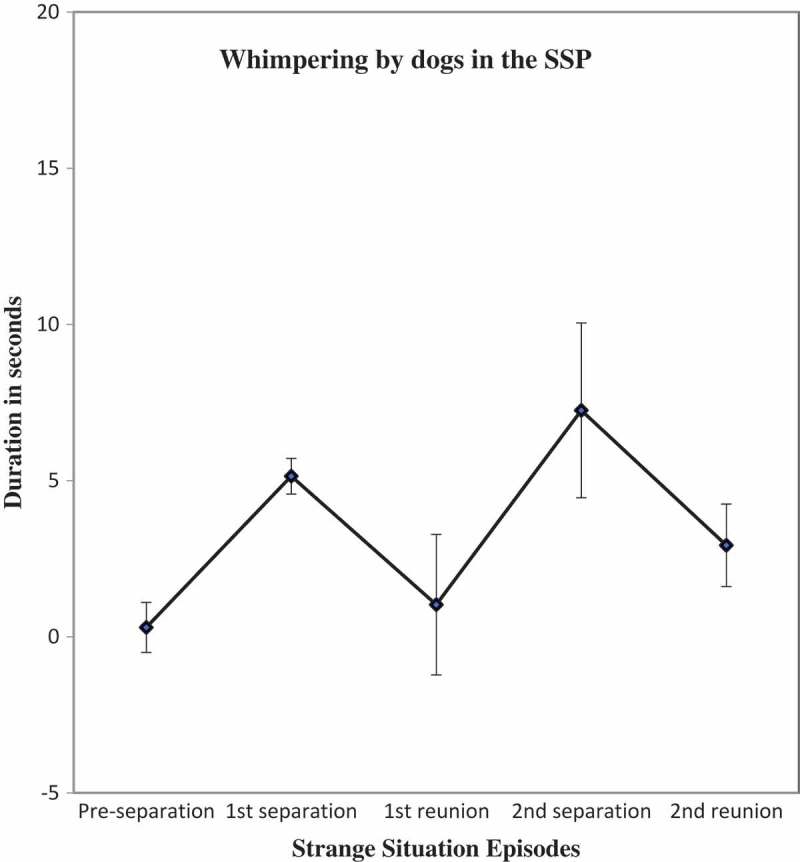
Duration of whimpering by dogs in the episodes of the strange situation (Mean ± SE).

**Figure 4.**
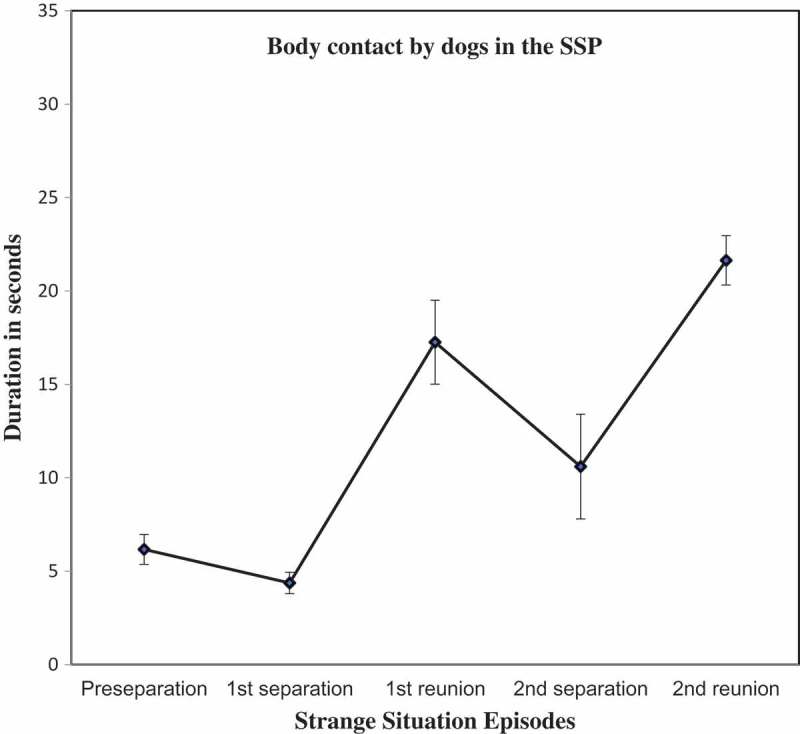
Duration of body contact with caregiver or stranger^1^ (Mean ± SE). ^1^ Duration of contact with caregiver is shown for the Preseparation and Reunion Episodes 1 and 2; Duration of contact with stranger is shown for the Separation episodes.

Results for analyses involving the secure base behavior coding partially confirmed that the procedures were sufficiently stressful to activate secure base behavior consistent with theoretical expectations. This is particularly important since the dogs in the study were adults and therefore might be less likely than puppies to display both attachment and exploratory behaviors. The changes in dog independent exploration, whimpering, and body contact across the episodes of the SSP are shown in Figures 2–4, respectively.

For the variable dog exploration, the repeated measures ANOVA showed a significant multivariate effect of episode (Wilk’s Lambda *F* (4, 55) 55.10, *p* < 0.001, partial *η* ^2^= 0.72). Planned, paired sample *t*-tests showed a significant decrease in exploration using the Holm-Bonferroni correction of alpha between the Pre-separation and Separation 1 episodes (*t* = 8.81, *df* = 58. *p *< .007, Cohen’s *d* = 1.52) and between Reunion 1 and Separation 2 (*t* = 2.27, *df* = 58, *d* = 0.39). However, the expected increase in exploration between Separation 1 and Reunion 1 (*t* = −.491, *df* = 58, *p* = .63, Cohen’s *d *=^ ^0.07) and between Separation 2 and Reunion 2 (*t* = −1.30, *df* = 58, *p* = 0.17, Cohen’s *d *= 0.24) were not significant, that is, the expected rebound in exploration during reunions did not occur. Due to the centrality of exploratory behavior to the secure base construct, we conducted post-hoc analyses of a second type of exploratory behavior, duration of independent play (defined as the dog playing with an object with no interaction with the caregiver for at least 5 s, eg chews on it, plays with it, or carries it around with toy in mouth). This variable did show the theoretically expected pattern, ie a high rate of play in the preseparation episodes, decreases in the caregiver’s absence followed by increases in the reunion episodes. The multivariate effect of episode in a repeated measures ANOVA was significant (Wilk’s Lambda (4,55) = 9.10, *p* < 001, partial *η* ^2^ = 0.42). Planned, paired comparison *t*-tests between all adjacent episodes were significant using the Holm-Bonferroni correction of alpha (Preseparation/Separation 1: *t* = 3.43, *df* = 58, *p* = .001, Cohen’s *d* = 0.49; Separation 1/Reunion 1: *t* = −3.469, *df* = 58, *p* = .001, Cohen’s *d* = 0.53; Reunion 1/Separation 2: *t* = 4.70, *df* = 58, *p* < .001, Cohen’s *d* = 0.66; Separation 2/Reunion 2: *t* = −2.88, *df* = 58, *p* = .005, Cohen’s *d* = 0.47).

For whimpering, the repeated measures ANOVA showed a significant main effect for episode (*F* (4, 55) = 5.11, *p* = .001, partial *η* ^2^ = .27). Planned, paired sample *t*-tests showed, as expected, significantly more whimpering in Separation1 in comparison to the Pre-separation period (*t* = −2.81, *df *= 58, *p* = .007, Cohen’s *d *= 0.51) and decreases in duration of whimpering between Separation 1 and the subsequent Reunion 1 (*t* = 2.68, *df* = 58, *p* = .009, Cohen’s *d* = 0.41) and between Separation 2 and Reunion 2 (*t* = 2.16, *df *= 58, *p *= .04, Cohen’s *d* = 0.35).

Preference for body contact with caregiver vs. stranger, which is a key aspect of the attachment construct, was tested in a repeated measures ANOVA that compared the relative duration of the dog’s physical contact with caregiver (Reunions 1 and 2) against the total duration with stranger (Separations 1 and 2). Results showed the predicted multivariate effect of episodes (*F* (1, 58) = 21.70, *p* < .001, partial *η* ^2^ = 0.27). Note that a direct comparison of the dogs’ contact with caregiver and stranger in the preseparation episodes was not included in the ANOVA because the behavior of the stranger is strictly constrained in the protocol for those episodes, rendering such a comparison spurious. Planned follow-up paired sample *t*-tests showed that dogs sought significantly more physical contact with the caregiver in the reunions than they did with the stranger in the adjacent separations, using the Holms-Bonferroni correction of alpha (Separation 1/Reunion 1: *t* = −3.80, *df *= 58, *p *< .001, Cohen’s *d* = . 0.61; Reunion 1/Separation 2: *t* = 2.12, *df* = 58, *p* = .04, Cohen’s *d* = .33; Separation 2/Reunion 2 (*t* = −3.44, *df *= 58, *p *< .001, Cohen’s *d* =^ ^0.57).

### Distribution of attachment classifications

Table 2 shows the distribution of classifications for the dogs in the sample in terms of both the 4-way classification system (groups A, B, C, and D) and the traditional Ainsworth A, B, and C system. For comparison, corresponding distributions of infants from two authoritative sources are also shown in the Table 2. Based on the 4-way classification system, the distribution of classifications in this study was 31 (61%) secure; 3 (6%) insecure-avoidant, 7 (14%) insecure-ambivalent, and 10 (20%) disorganized. This distribution is generally consistent with that reported in a meta-analysis of 15 low-risk, US infant–mother samples (van IJzendoorn, Schuengel, & Bakermans-Kranenburg, 1999), although the avoidant group appears to be somewhat under-represented. When the D cases were redistributed into their best-fitting ABC groups, the distribution was more similar to Ainsworth et al.’s (1978) results based on four US samples.

**Table 2.** Frequency distribution of dog attachment classifications compared to normal US infant samples.

Classification group	Dog “4-way” classification (A, B, C, and D)^a^*N*(%)	Infant “4-way” classification (A, B, C, and D) (van IJzendoorn, 1995)^b^*N*(%)	Dog “ 3-way” classification (A, B, and C)^a,c^*N*(%)	Infant “3-way” classification (A, B, and C) (Ainsworth et al., 1978)^c,d^*N*(%)
Secure (B)	31 (61%)	*62%*	34 (66%)	*66%*
Avoidant (A)	3 (6%)	*15%*	7 (14%)	*22%*
Ambivalent (C)	7 (14%)	*9%*	10 (20%)	*13%*
Disorganized (D)	10 (20%)	*15%*	–	__

^a^ *N* = 51 dog–caregiver dyads; unclassified cases excluded.

^b^ *N* = 2,104 infant–mother dyads; based on normative samples from 15 studies.

**^c^**Dogs assigned to the insecure-disorganized classification were re-assigned to their best-fitting (alternative) A, B, or C group.

^d^ *N* = 106 infant–mother dyads; based on normative samples

### Relationships among attachment security, control variables, and caregiver reassuring presence

The intercorrelations among the study variables are shown in Table 3. As predicted, caregiver supportive presence was negatively related to insecure classification. The remainder of the bivariate correlations were not specifically predicted: Female caregivers rated their dogs as more active/excited (*r *= .44, *n* = 49, *p* < .002). Active/excitable dogs were significantly less likely to be judged as secure (*r* = .36, *n* = 49, *p* = .011) and also were more likely to be younger (*r* = .36, *n *= 50, *p* = .009). Older caregivers rated their dogs as more insistent/goal-determined (*r* = .367, *n* = 49, *p* < .009); male dogs also were rated as more insistent (*r* = −.306, *n *= 49, *p* < .032). Insistent dogs were rated as less nervous (*r* = −.348, *n* = 49, *p* = .014). When all of the variables above were entered into the GLM, only Caregiver Reassuring Presence (Beta = .415, Wald Chi-square = 5.36, *df* = 1, *p* = .021) and active/excitable dog personality (Beta = −.975, Wald Chi-square = 4.417, *df* = 1, *p* = .030) remained as significant predictors of secure vs. insecure attachment. The means for the secure and insecure groups for these two variables are shown in Table 4, as are the means for the three insecure groups, presented for heuristic value.

**Table 3.** Bivariate intercorrelations among study variables.^1^

Demographic variables					MCPQ-R Scales					Caregiver and attachment variables	
	1 Owner gender	2 Owner age	3 Dog Gender	4 Dog age	5 Active	6 Obedient	7 Insistent	8 Nervous	9 Reserved	10 Supp Presence	11 Secure-Insec
1	-										
2	.049	-									
3	.247^+^	.162	-								
4	.102	−.016	−.168	-							
5	.438**	−.073	.008	−.319*	-						
6	.194	.190	.147	−.089	.093	-					
7	.193	.367**	−.306*	-.092	−.061	.029	-				
8	−.201	−.183	.172	.028	.014	−.009	−.348*	-			
9	−.126	.023	−.157	.041	−.100	−.061	−.030	.222	-		
10	.085	.032	.197	.007	.156	.075	.102	−.044	−.031	-	
11	.193	−.004	−.058	−.155	.360*	.098	.020	−.149	−.226	−.363*	-

***N* = 49** due to missing data. ^+^ *p* < .10; *p < .05; **p < .01.

**Table 4.** Means and standard deviations for Caregiver Reassuring Presence (CRP) and dog active/excitable temperament (MCPQ-R).

Attachment classification	CRP Mean (sd) (*N* = 50)^a^	Active/excitable Mean (sd) (*N* = 50)
Secure (*N* = 31)	4.56 (1.87)	−0.18 (1.02)
Insecure (*N* = 19)	2.94 (1.88)	0.50 (0.60)
**Insecure classifications**		
Avoidant (*N* = 3)	1.50 (.87)	.40 (.96)
Ambivalent (*N* = 7)	3.86 (1.94)	.57 (.69)
Disorganized (*N* = 9)	2.95 (1.94)	.49 (.46)

^a^1 case dropped due to missing MCPQ-R data.

## Discussion

The results of this study indicate the feasibility of using the “gold standard” of developmental psychology – Ainsworth’s Strange Situation and classification system – to delineate differences in attachment security in dog–caregiver attachment (Ainsworth et al., 1978). First, carefully following the order and timing of episodes as designed by Ainsworth resulted in normative shifts in the frequencies of behavior, paralleling those shown by infants, ie high levels of exploration of the room prior to separation, distress and search in separation, and close physical contact with the caregiver during reunion (at levels higher than those with the stranger in separation). Consistent with Bowlby’s ethological attachment theory and the infant–parent literature, the behavior of most dogs conformed to the secure pattern. This is characterized by use of the caregiver as a secure base in a novel environment, search during separation, immediate interest in regaining proximity and contact following separation, and resumption of exploration thereafter. This finding is consistent with that of other investigators who described a secure pattern as more common but who did not formally define or validate the construct (Rehn, Handlin, Uvnäs-Moberg, & Keeling, 2014; Rehn & Keeling, 2011; Taggert, 2010; Topál et al., 1998).

This is the first investigation to identify the insecure attachment patterns in dogs (avoidant, ambivalent and disorganized/disoriented) following Ainsworth et al. (1978) and Main & Solomon’s (1990) criteria. Overall, the proportion of insecure attachments was comparable to infant–mother samples, although the distribution of the three insecure classifications differed somewhat. In particular, the avoidant group appeared to be comparatively under-represented. In previous dog research, attachment insecurity has been defined only in terms of manifest anxiety (cf. Taggert, 2010)

Results also showed that the secure and insecure groups differed with respect to the caregiver’s reassuring presence when the dog was approached by a “threatening stranger” in a previous laboratory session. The association of caregiver sensitivity with the secure classification is a consistent finding in the human literature (van IJzendoorn, 1995) and, indeed, is central to understanding security as a reflection of the history of interaction between parent and child (Solomon & George, 2016). The demonstration of this association for dogs and their caregivers supports the argument that the dog attachment groups reflect differences in underlying relationship rather than mere behavioral homology to human infants. In support of our findings linking CRP to dog security, Schöberl (2017) reports a correlation between a calm, “friendly” caregiver response to their dog in the presence of the “threatening stranger” and low heart rate and aggression on the part of the dog.

In another report involving this sample Schöberl et al. (2016) found that secure as opposed to insecure dog attachment was associated with lower cortisol secretion in the SSP and a play situation with the caregiver, suggesting that the secure dogs found these situations less stressful. In addition, security was associated with a tendency for cortisol to increase over baseline when the dog faced the threatening stranger in the caregiver’s absence. Both sets of findings would be expected if the caregiver were a “secure base” for the dog. The findings are also consistent with Gacsi’s et al’s (2013) report of greater modulation in dogs’ heart rate when faced with a threat in the caregiver’s presence, which they too interpreted as evidence of a “safe haven” effect (Gácsi et al., 2013).

This study represents the first systematic evidence of disorganized attachment patterns in dog–human relationships. Exemplars of all of the major categories of disorganization in infants (Main & Solomon, 1990) were evident in this sample. The most common of these were simultaneous approach-avoidance conflict, fearful shying away or hiding from the caregiver, marked stereotypies, and extended tonic immobility in the absence of an obvious threat. The latter is often interpreted in infants as akin to a dissociative state. These behaviors are well known in the ethological literature as responses to various levels of threat or fear (Hinde, 1966). In the case of human infants such behaviors led to the hypothesis that the parents of such infants were in some way alarming, resulting in an “irresolvable” conflict about approaching the parent under stress (Main & Hesse, 1990). This has been borne out in studies that show a close link between attachment disorganization and seriously disturbed parenting in both high-risk and normative samples (Lyons-Ruth & Jacobvitz, 2016). Other developmental pathways to disorganized infant attachment include major or repeated separation from the caregiver under adverse conditions (Solomon & George, 1999) and regulatory difficulties or genetic predispositions to such difficulties in interaction with insensitive caregiving (Luijk et al., 2011; Padrón, Carlson, & Sroufe, 2014; Spangler, 2011). Thus while disorganized behavior in the dog may prove to be a useful indicator of problematic care, more research is required to understand fully its antecedents or to rely on it as a marker of risk (Grandqvist et al., 2017).

Results also showed an effect of the dog’s temperament on his or her attachment security. Caregivers’ with secure dogs rated them as significantly less active/excitable than insecure ones. Conceivably, this correlation reflects the fact that more active and excitable dogs require more oversight or possibly stricter training methods which could lead to the use of harsher or more inconsistent methods of control, either of which might affect the dog’s eagerness to approach and seek physical contact or ability to be settle quickly after separation. However, this causal pattern would be expected to affect other ratings as well, such as for obedience or nervousness or caregivers’ supportive presence, which was not the case here. Furthermore, caregiver ratings cannot be considered pure assessments of inherited or temperamental traits (Cassidy & Koback, 1988) and we cannot determine the direction of effects. It would be worthwhile to confirm the current findings using more behaviorally based measures of temperament (Fratkin et al., 2013) or to compare attachment classifications among breeds known to differ on activity level.

To our knowledge, this is the first demonstration of the avoidant attachment pattern in dogs, although it was rare (*N* = 3) or often accompanied by substantial signs of behavioral conflict or disorganization (*N* = 4). With or without concomitant signs of disorganization, avoidance was associated with low caregiver sensitivity to the dog’s distress. The avoidant pattern in infants has long been understood to reflect a defensive or self-regulatory strategy that allows the infant to maintain some distance from a caregiver who habitually discourages physical contact (Ainsworth et al., 1978; Main, 1981). Evidence for this in infants is found in studies showing that, though outwardly calm, the avoidant infant is actually experiencing increased heart rate and cortisol levels in the SSP comparable to or higher than that of secure infants (Spangler & Grossmann, 1993; Sroufe & Waters, 1977). Thus, avoidance appears to depend on a degree of cognitive, though not necessarily conscious, control of behavior. Infant macaques do not show avoidance following separation from the mother (Ainsworth, Bell, & Stayton, 1971); however this pattern has been demonstrated in captive, juvenile chimpanzees, albeit at low frequencies (Van Ijzendoorn et al., 2008). The existence of an avoidant pattern outside the primate order raises intriguing questions regarding underlying mechanisms that cannot be answered at present. For example, do avoidant dogs experience increased heart rate during reunion as babies do despite the appearance of calm disinterest in the caregiver? If so, what are the underlying mechanisms that permit them to direct attention and behavior away from the caregiver despite the activation of an instinctual response compelling approach and proximity-seeking? These capabilities are not inconsistent with the relatively complex cognitive processing and control of behavior in the dog demonstrated in recent studies, but go beyond what previously has been established (Bray, MacLean, & Hare, 2014).

As the first of its kind, it is important to consider the limitations of this study. The adaptation of Ainsworth’s classification methods involved collaboration between investigators with different backgrounds and familiarity with the infant attachment measure and dogs. Not surprisingly, agreement between judges was sometimes elusive. A satisfactory level of inter-coder agreement was reached, but it was necessary to “agree to disagree” on 13 (22%) of the sample. Some of these cases (*N* = 8; 14%) were left unclassified because the dog’s behavior was in some way odd or ambiguous but not clearly disorganized.

The demarcation of the insecure-ambivalent group also was problematic. Ultimately, 5 of the 13 cases above were assigned to that classification, on the basis of only one of the two judges’ opinions. Ambivalent infant classifications tend to be rare in most infant–mother samples (van IJzendoorn, 1995) and the number of ambivalent dogs found here is consistent with that observation. In contrast, investigators of attachment in dogs have reported an anxious pattern (ie little exploration prior to separation, strong search and/or protest during separation, and extensive contact maintaining during separation) to be relatively common among companion dogs (Fallani et al., 2006; Topál et al., 1998). The criteria for Ainsworth’s ambivalent infant group, the group which best corresponds to an “anxious” pattern, include in addition to the aforementioned behaviors: “contact resistance” (eg irritable rejection of contact or toys, “tantrum” behavior, wriggling in contact). Although a number of dogs in the current study displayed persistent anxiety, signs of resistance or aggression were rare and subtle, making classification more difficult. Presumably, in the course of domestication there was strong selection against any tendency in the dog to “punish” an inconsistently responsive caregiver. A series of recent experiments of equivalently raised and kept wolves and dogs indicates that there was a strong selection during domestication toward accepting dominance and hierarchies (Range, Ritter, & Viryani, 2015) and toward being submissive to humans (Range et al. pers. comm.). Furthermore, caregivers are prone to relinquish aggressive dogs, lowering the chance that we would see this behavior in a self-selected sample, such as ours. It is likely that further work will be required to differentiate anxiety about the caregiver’s whereabouts from signs of a generally anxious temperament (cf. Belsky & Rovine, 1987). In this regard it is important to note that although caregivers of secure dogs were significantly higher in sensitivity than those of avoidant and disorganized dogs, caregivers of secure and ambivalent dogs were more similar. This may indicate that not all of the dogs placed in group were truly “insecure.”

We made additional decisions to accommodate the dog’s behavioral repertoire that also may have limited the accuracy of classifications. For example, similar to other investigators (eg Prato-Previde et al., 2003) we observed that many dogs followed the unfamiliar playmate to the door when she left the testing room shortly after the dog and caregiver had reunited. We discounted this as a sign of insecurity, as it might be for infants (Main & Solomon, 1990), because of its ubiquity. Others appear to have interpreted indications of dogs’ attraction to the playmate as a potentially important sign of lack of exclusivity of attachments in the species or at least an experimental confound (Palmer & Custance, 2008; Rehn et al., 2013). This is plausible since many dogs have been bred for friendliness toward people in general (sledge dogs, modern hunting dogs), potentially constraining exclusive dyadic attachment. However, when investigators have looked more closely at dogs’ behavior in the SSP or counterbalanced episodes to equate for time with the stranger, preference for the caregiver usually emerged more clearly. Conceivably, dogs’ strong interest in the playmate has evolutionary origins in the group orientation of wolves, in contrast to the more exclusive orientation of primate offspring toward a single caregiver.

We also gave less weight in assigning classifications to dogs’ exploration and object play than would be the case for infants. This was based on our direct observation of variations along this dimension during the SSP (eg two dogs appeared to settle in for a nap after greeting the caregiver in the second reunion). The behavior variable “independent exploration” – which focused on literal exploration of the environment – was selected a priori to establish the suitability of the SSP protocol, but results showed that it did not perform as expected: High rates of exploration before the first separation decreased considerably over the course of the procedure, which precluded finding differences exploration between later episodes. However, *post hoc* analyses of the variable “independent play” by dogs, in actuality a closer equivalent to infant play in the SSP, did show the expected significant peaks in the episodes involving the caregiver and declines in his or her absence. Thus, although the dogs in this sample were well beyond puppyhood, it would appear that the protocol elicited enough stress to activate the attachment system and differential patterns of exploration in the presence of the caregiver vs. strangers. However, Fallani et al. (2006) found the amount of play in the SSP also was related to the age of the dog. Replication of our procedures with puppies or younger dogs, and/or more novel toys would be helpful in further establishing the validity of the classifications. Additional improvements to this system also may be pursued through the study of larger samples and sample selection on the basis of breed, temperament, age, or placement history. Crucially, this exploratory study is further limited by the absence of observations of dog–caregiver interaction over time and in more naturalistic contexts. The strength of Ainsworth’s system lay in the careful empirical demonstration of the specific interaction histories corresponding to placement in the various classification groups. The pitfalls of bypassing this systematic approach are illustrated by subsequent failures to find theoretically predicted continuity in the attachment classifications of 3-year-old children based on a system extrapolated from infant patterns and general developmental knowledge but without extensive observation of mother–child interaction in other contexts (Solomon & George, 2016).

Nevertheless, the comparability of the human and dog attachment patterns in behavior and in physiology (Schöberl et al., 2016) found in this study is consistent with the hypothesis that the canid and primate attachment behavior systems are organized along generally similar lines. Indeed, recent research suggests that, in contrast to earlier reports (Topál et al., 2005), wolves also show attachment behavior to the latter human caregivers in the SSP when this is assessed at 3–7 weeks of age (Hall, Lord, Arnold, Wynne, & Udell, 2015). Adduced to mounting evidence of the general comparability of neurophysiological organization and hormonal control underlying mammalian social behavior (Berns, Brooks, & Spivak, 2015; Goodson, 2005; O’Connell & Hofman, 2012), these results suggest that the organization of the attachment system may be a highly conservative evolutionary attribute. In fact, the “social network in the brain” has turned out to be the most evolutionary conservative structure of the brain and of the body in general (Goodson, 2005; O’Connell & Hoffman, 2012). Additionally, oxytocin has complex, context-specific functions in social behavior, for humans and dogs, including that between dog and human (Turcsán, Miklósi, & Kubinyi, 2017). This does not preclude the likelihood that future research will reveal species’ and individual differences in factors, such as exclusivity, longevity of bonds, developmental timing, or behavioral flexibility under non-optimal caregiving conditions or that some species, such as the domestic cat (Potter & Mills, 2015) will show attachment behavior only in quite limited circumstances.

Although replication of these exploratory findings is necessary, the application to the dog of Ainsworth’s procedures for capturing individual differences in attachment security potentially opens up new territory for comparative and anthrozoological studies. With this standardized approach to the description of relationships, it may soon be possible to address more precisely empirical questions pertaining to the development of dog–caregiver attachment, the effects of separation of dogs from caregivers for training and other purposes, relationship disruption and trauma, dog training regimes, and caregiver styles. These matters are of central importance to dog caregivers and handlers, as well as to developmental biologists; they may also provide comparative and evolutionary insights to investigators of human relationships. Additionally, dogs increasingly are used as therapeutic adjuncts, for example in intervention with autistic and mentally disturbed children and adults, for comfort in health care settings, and to improve learning outcomes among normal children (Nimar & Lundahl, 2015; Payne et al., 2015). Assessment of the security of dog attachment in these settings may in some cases explain the success or failure of such interventions and be used to monitor improvement in the social skills of dogs’ caregivers.

## Notes

1. I am indebted to Robbie Duschinsky who is working with the Bowlby Archives at the Wllcome Trust for the insight and the quotation from Bowlby (1961).

2. The formula for the Holm-Bonferroni is: Target Alpha Level/ n-rank number of pair (by degree of significance) +1 (*http://www.statisticshowto.com/holm-bonferroni-method/*).

3.
http://www.socialstatistics.com/effectsize/Default3aspx.
